# Multiparametric MRI and artificial intelligence for non-invasive HER2 assessment in breast cancer: a comprehensive review

**DOI:** 10.3389/fmed.2026.1893638

**Published:** 2026-08-07

**Authors:** Yao Zhang, Shiyi Cheng, Qun Yang, Yun He, Xiulan Zhang, Guanghai Ji

**Affiliations:** 1 Department of Radiology, The First Affiliated Hospital of Yangtze University, Jingzhou, Hubei Province, China; 2 Department of Medical Imaging, School of Medicine, Yangtze University, Jingzhou, China; 3 Department of Anatomy, School of Medicine, Yangtze University, Jingzhou, China

**Keywords:** breast cancer, deep learning, human epidermal growth factor receptor 2 (HER2), multiparametric MRI, radiomics

## Abstract

**Objective:**

To systematically evaluate the utility of multiparametric MRI (mpMRI) combined with radiomics and artificial intelligence (AI) for non-invasive assessment of human epidermal growth factor receptor 2 (HER2) status in breast cancer.

**Methods:**

Following the PRISMA 2020 guidelines, we searched PubMed, Web of Science, and Embase for studies published between January 2015 and December 2025 (last search: 27 June 2026). After duplicate removal using Zotero, two reviewers independently screened the records. Thirty-four studies were included and assessed using PROBAST, with the 16 radiomics studies additionally evaluated using the RQS. We qualitatively synthesized evidence on conventional MRI, diffusion imaging (DWI/ADC, IVIM, DKI, and DSI), DCE-MRI, metabolic MRI, and radiomics/AI approaches without performing a meta-analysis.

**Results:**

mpMRI sequences provide complementary insights into HER2 status. Advanced diffusion techniques (DKI: *p* = 0.003; DSI: AUC range, 0.700–0.721) and DCE-MRI radiomics provide predictive value by capturing microstructural and dynamic tumor characteristics. Multiparametric radiomics incorporating peritumoral features achieved AUCs of 0.84–0.923 for three-level HER2 classification, while radiomics and deep learning distinguished HER2-low from HER2-zero tumors with AUCs up to 0.892. Machine learning and deep learning models demonstrated moderate cross-center generalizability (external AUC, 0.66–0.84). However, variability in reference standards, retrospective study designs, and limited external validation remain major limitations.

**Conclusion:**

mpMRI combined with radiomics and AI demonstrates promising value for HER2 assessment, with internal testing often AUCs exceeding 0.90 but less consistent on external validation. It complements rather than replaces biopsy by capturing intratumoral heterogeneity and supporting treatment decisions. Future priorities include prospective multicenter validation, methodological standardization, and radiopathogenomic integration.

## 1 Introduction

Breast cancer is the most common malignancy in women, with over 2.3 million new cases annually, representing 11.7% of all cancers (1). Human epidermal growth factor receptor 2 (HER2) is a key molecular biomarker whose expression directly guides treatment decisions and prognosis. The DESTINY-Breast04 trial (2) showed that trastuzumab deruxtecan (T-DXd) markedly improves survival in HER2-low advanced breast cancer, prompting a shift from a binary to a three-tier HER2 classification: HER2-zero, HER2-low, and HER2-positive. Current clinical assessment relies on immunohistochemistry (IHC) and *in situ* hybridization (ISH), which are invasive and prone to sampling error due to intratumoral heterogeneity (11–18%) (3). Multiparametric MRI (mpMRI) combined with radiomics and artificial intelligence (AI) provides a non-invasive, radiation-free complementary method for HER2 evaluation.

## 2 Methods

This systematic review followed the PRISMA 2020 guidelines. We searched PubMed, Web of Science, and Embase for studies published between 1 January 2015 and 31 December 2025, with the last search conducted on 27 June 2026. The full search strategies are provided in Supplementary Table S1. Only studies published within this period entered the PRISMA workflow; records published from 1 January 2026 onward were not screened or included in the quality assessment.

Eligible studies were original peer-reviewed breast cancer studies that reported non-invasive HER2 assessment using MRI/mpMRI, radiomics, machine learning, or deep learning for binary or three-tier classification, included performance metrics such as the AUC, enrolled at least 20 patients, and were available as full-text articles in English. We excluded case reports, reviews, non-breast, non-MRI, or non-HER2 studies, studies with insufficient data or duplicate cohorts, and non-English full-text articles.

**Figure 1:**
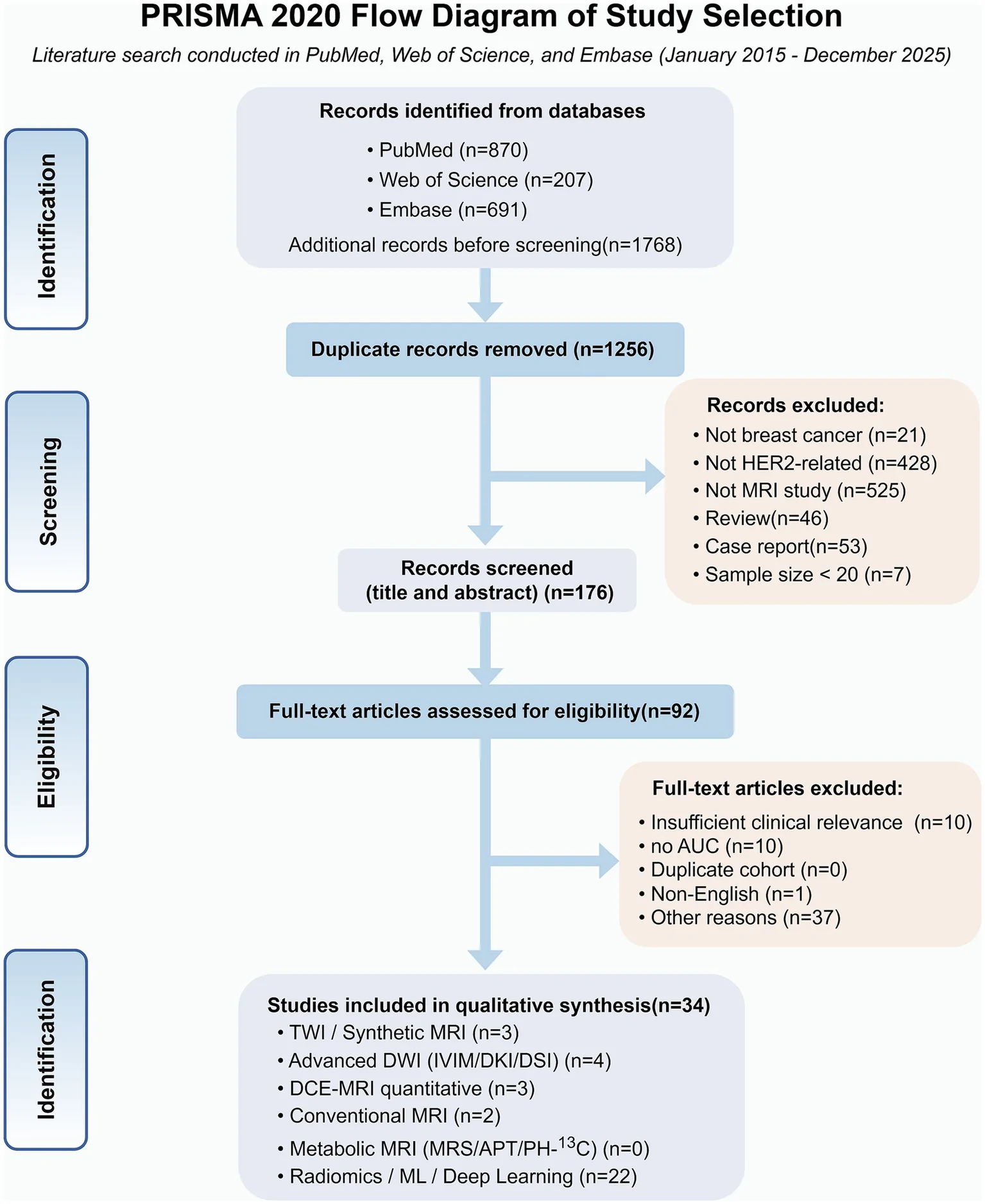
PRISMA 2020 flow diagram of the literature search and study selection process. Records were identified through PubMed, Web of Science, and Embase for studies published between January 2015 and December 2025. Reasons for exclusion at each stage are shown in the corresponding boxes. PRISMA, Preferred Reporting Items for Systematic Reviews and Meta-Analyses.

After duplicate removal in Zotero, two reviewers (Y. Zhang and G. Ji) independently screened titles, abstracts, and full texts. Any disagreements were resolved through discussion or, when necessary, by a senior reviewer (X. Zhang). The entire screening process is documented in *PRISMA_ledger.xlsx* (Figure 1).

Thirty-four studies remained after full-text reassessment (Sheet 6 of *PRISMA_ledger.xlsx*) and were included in the data synthesis and quality assessment. These studies met all prespecified eligibility criteria among 92 retrievable full-text articles, of which 58 were excluded. They do not correspond exactly to the reference list, which also includes background reviews, landmark studies published before 2015, and *post hoc* contextual citations that were not part of the PRISMA screening or PROBAST/RQS evaluation.

All 34 included studies were assessed using PROBAST. The Radiomics Quality Score (RQS) was additionally applied to the 16 radiomics studies, whereas the remaining 18 non-radiomics studies were not applicable. All assessments were performed independently by two reviewers and finalized by consensus (Supplementary Tables S2, S3). Because of methodological heterogeneity, the findings were synthesized qualitatively without meta-analysis.

**Figure 2:**
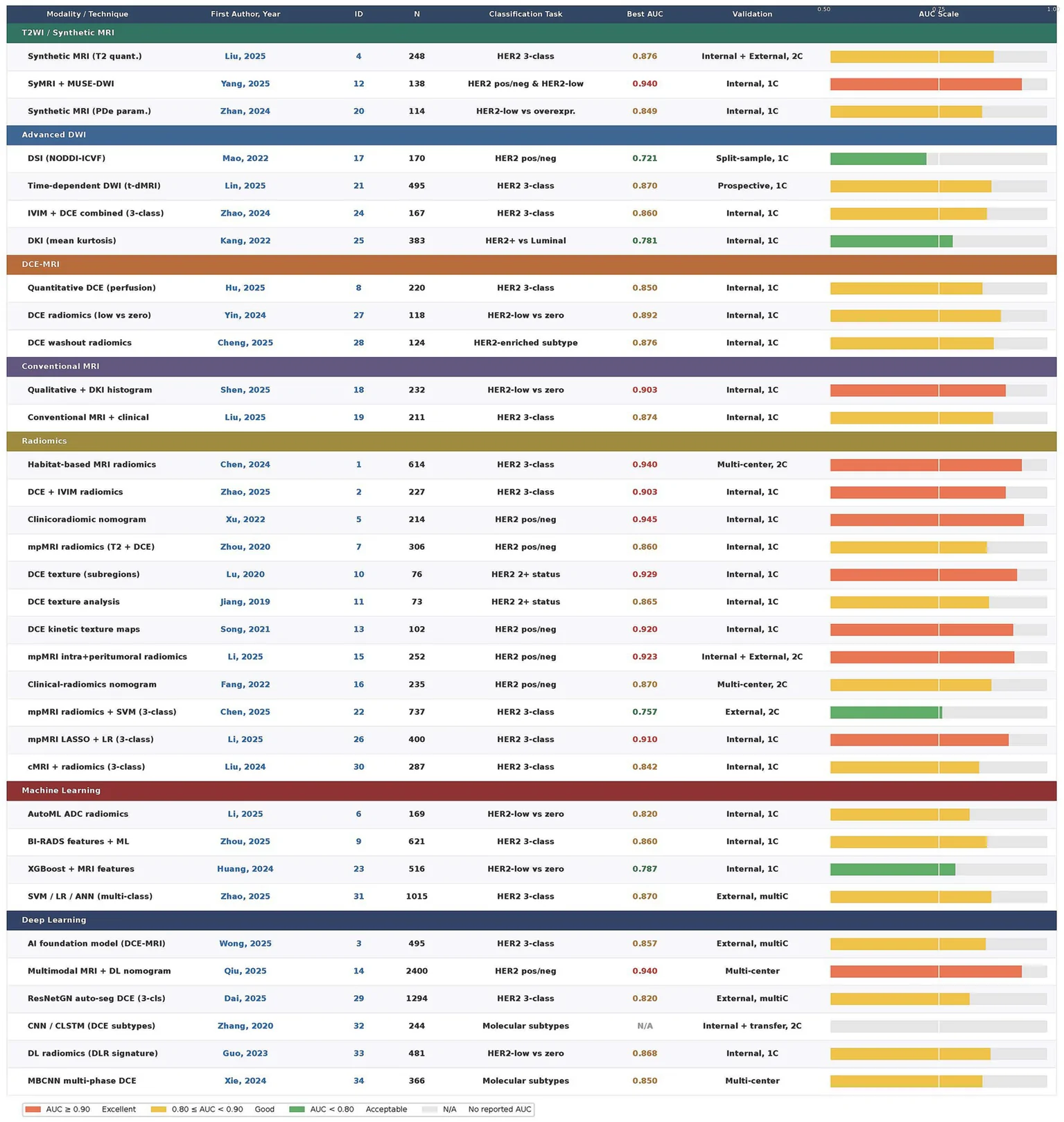
Summary of MRI-based performance for predicting HER2 status across different imaging techniques. Studies were grouped by imaging modality. In the “AUC scale” column, the colored bar indicates the highest reported AUC, and its length reflects performance on a 0.50–1.00 scale. Colors represent diagnostic performance levels: Red, excellent (AUC ≥ 0.90); amber, good (0.80 ≤ AUC < 0.90); and green, acceptable (AUC < 0.80). AUC, area under the receiver operating characteristic curve; DCE-MRI, dynamic contrast-enhanced MRI; DKI, diffusion kurtosis imaging; DWI, diffusion-weighted imaging; IVIM, intravoxel incoherent motion; MRI, magnetic resonance imaging; N, sample size; SVM, support vector machine; 1C/2C, single-center/dual-center. See Supplementary Table S4.

**Figure 3:**
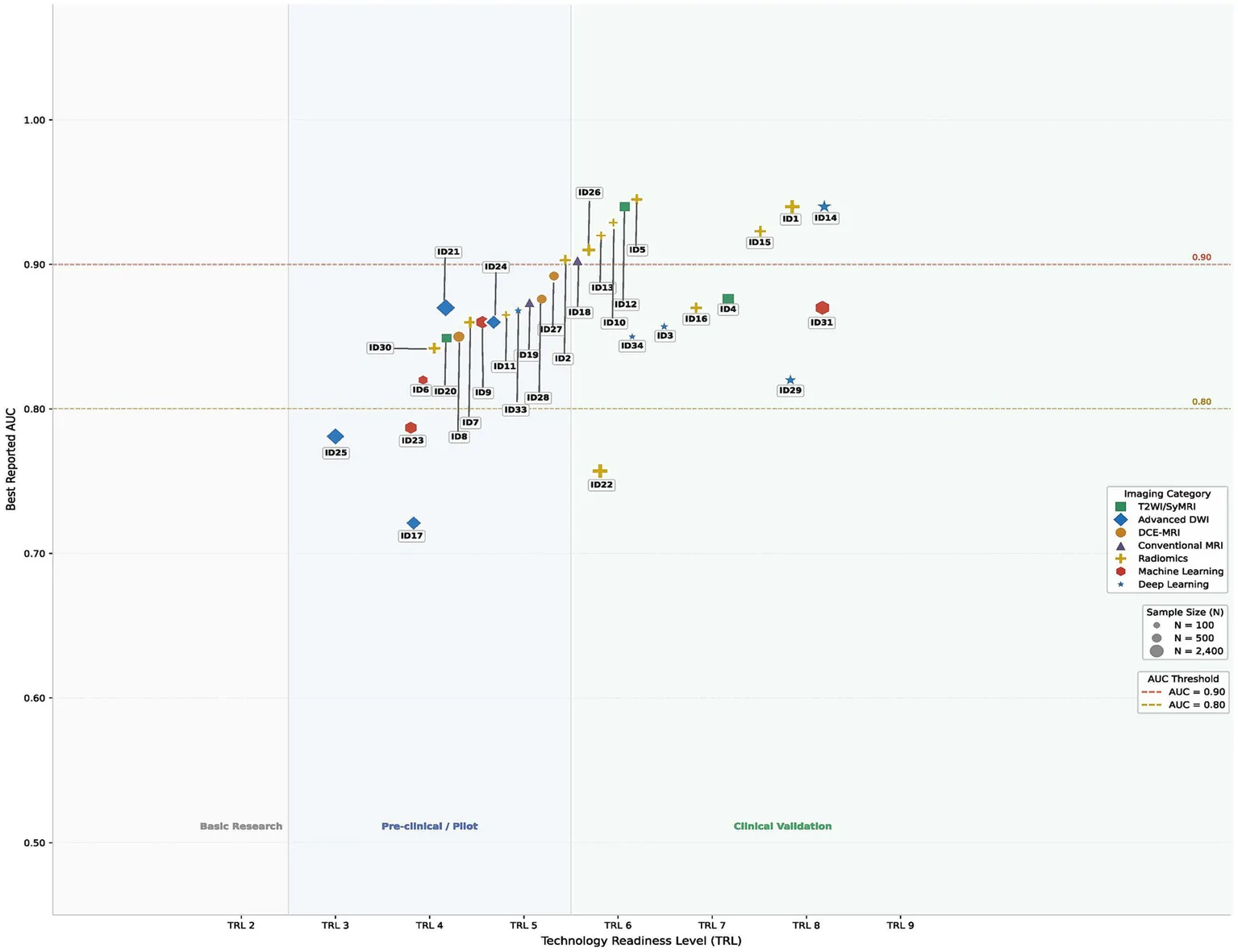
Technology readiness level (TRL) roadmap for MRI-based HER2 assessment in breast cancer. Each bubble represents an included study with a reported predictive AUC (*n* = 33 of 34). TRL values (2–9) were assigned to all included studies according to the prespecified criteria described in Section 2. The x-axis represents the assigned TRL based on study design and validation strategy, whereas the y-axis indicates the highest reported AUC. Bubble size is proportional to sample size (*N*^2/5^). Numeric labels correspond to study IDs in Figure 2, with leader lines connecting each label to its corresponding bubble. Background shading represents three developmental phases: basic research (TRL 2), preclinical or pilot validation (TRL 3–5), and clinical validation (TRL 6–9). One study without a reported predictive AUC (ID 32; Zhang et al., 2020; accuracy metrics only) was excluded from the bubble plot and is summarized in Figure 2.

For each included study, we extracted performance metrics, including the highest reported AUC, together with the technology readiness level (TRL). Figures 2, 3 summarize only the 34 studies included after full-text review. References cited for background, methodology, or contextual discussion were not included unless they satisfied the eligibility criteria and passed full-text assessment.

## 3 HER2 assessment: pathological classification and clinical significance

According to the 2023 ASCO/CAP guideline update (4), HER2 IHC scoring is defined as: IHC 0 (no staining or ≤10% weak incomplete membrane staining), IHC 1+ (>10% incomplete membrane staining), IHC 2+ (>10% complete weak/moderate staining), and IHC 3+ (>10% complete strong staining), with IHC 2+ requiring ISH confirmation. HER2 is thus stratified as HER2-zero (IHC 0), HER2-low (IHC 1+ or IHC 2+/ISH−), and HER2-positive (IHC 3+ or IHC 2+/ISH+), with HER2-low accounting for 55%–60% of cases (5) and showing therapeutic benefit from antibody-drug conjugates (2).

HER2 expression exhibits pronounced spatial and temporal heterogeneity, directly impacting pathological accuracy: intratumoral heterogeneity occurs in 11–18% of cases (3), while intertumoral discordance between primary and metastatic sites affects 5%–12% (6), with greater divergence in distant metastases. Therapeutic pressure can further amplify these differences. Consequently, single-site biopsy is prone to sampling error, risking HER2 misclassification and suboptimal treatment, underscoring the inherent limitations of pathology as a reference standard. Consistent with this concern, biomarker profiles in residual disease may differ from those in pretreatment biopsies after neoadjuvant chemotherapy. Large cohort studies have shown that HER2-positive tumors are particularly prone to biomarker conversion (7), further supporting the reassessment of residual disease rather than relying uncritically on pretreatment labels in supervised modeling.

## 4 Conventional MRI sequences in HER2 assessment

Conventional MRI sequences remain fundamental to breast imaging. T1-weighted imaging (T1WI) primarily provides an anatomical reference for DCE-MRI and has limited direct relevance to HER2 status. In contrast, T2-weighted imaging (T2WI) and BI-RADS descriptors have been associated with three-tier HER2 classification. BI-RADS-based models show modest discriminatory performance, with AUCs of 0.79 for HER2-zero versus HER2-non-zero tumors and 0.69 for HER2-low versus HER2-positive tumors (8). Qualitative MRI features have also achieved an AUC of 0.763 for distinguishing HER2-low from HER2-zero disease (9). Quantitative approaches generally yield better performance, including SyMRI T2 with an AUC in external validation of up to 0.837 (10), combined conventional MRI models with AUCs in test cohorts of 0.845 and 0.805 (11), and SyMRI–MUSE-DWI nomograms with AUCs of 0.940 and 0.810 (12). These findings suggest that conventional MRI provides a structural foundation that can be further leveraged by integrated radiomics and AI frameworks.

## 5 Diffusion imaging techniques in HER2 prediction

### 5.1 Conventional DWI and ADC

Diffusion-weighted imaging (DWI) quantifies microscopic water motion, with the apparent diffusion coefficient (ADC) reflecting cellular density, membrane integrity, and extracellular space (13). ADC is b-value dependent: low b-values (50–100 s/mm^2^) incorporate perfusion effects, whereas higher b-values (800–1,000 s/mm^2^) more accurately reflect true diffusion. Mean ADC alone has limited ability to discriminate HER2 status, with a validation AUC of 0.568 (14). However, intratumoral diffusion heterogeneity remains informative. Compared with mean ADC, histogram-based analyses incorporating percentiles, kurtosis, and skewness better capture diffusion heterogeneity and improve HER2 subtype prediction, achieving an AUC of 0.87 for distinguishing HER2-positive from HER2-negative tumors (15).

ADC-based radiomics distinguished HER2-zero from HER2-low tumors, achieving a validation AUC of 0.75 (16). Li et al. (17) reported a testing AUC of 0.713 using ADC radiomics, which increased to 0.915 in the testing cohort and 0.837 in the external validation cohort after incorporating 3-mm peritumoral features. Chen et al. (18) applied mpMRI radiomics incorporating DWI in 737 patients and achieved external validation AUCs of 0.757 and 0.754. Liu et al. (19) reported a test AUC of 0.91 for differentiating HER2 overexpression from non-overexpression, while Huang et al. used machine learning with ADC to distinguish HER2-zero from HER2-low tumors, yielding a validation AUC of 0.787 (20).

In the neoadjuvant setting, ΔADC has shown potential for predicting pathological complete response (21), though its specific utility in HER2-positive disease remains uncertain, as targeted therapies primarily act through signaling blockade rather than direct cytotoxicity.

### 5.2 Advanced diffusion techniques (IVIM, DKI, DSI, and combined models)

A key limitation of conventional ADC is its reliance on a monoexponential model, which does not fully separate true water diffusion from microvascular perfusion. Because these processes reflect distinct aspects of tumor biology, combining them into a single parameter can reduce biological specificity, particularly in highly vascularized breast tumors.

IVIM separates the diffusion signal into true diffusion (D) and perfusion components (D* and f), providing greater biological specificity than conventional ADC. HER2 overexpression promotes angiogenesis via VEGF upregulation, theoretically elevating f and D* values. Among included predictive studies, Zhao et al. (22) reported significant differences in IVIM f and DCE-MRI Ktrans across HER2-zero, HER2-low, and HER2-positive groups (*p* < 0.05), supporting functional imaging-based HER2 stratification. However, IVIM parameter fitting remains technically demanding, limiting reproducibility across institutions.

DKI extends diffusion analysis by capturing non-Gaussian water diffusion to assess microstructural complexity. Among the included predictive studies, Kang et al. (23) reported significantly higher mean kurtosis (MK) in HER2-positive tumors than in HER2-negative tumors (1.12 ± 0.18 vs. 0.98 ± 0.21, *p* = 0.003). MK was positively correlated with Ki-67, suggesting that it reflects proliferative activity rather than HER2 expression alone. In the same study, kurtosis also differed across molecular subtypes, with lower values in HER2-positive than in luminal tumors, achieving an AUC of 0.781 for distinguishing HER2-positive from luminal tumors. These findings further suggest that DKI-derived kurtosis is more closely associated with subtype biology than with HER2 expression alone. Nevertheless, substantial heterogeneity across DKI studies, including differences in study populations, metric definitions, and HER2 classification strategies, highlights the need for greater methodological standardization before DKI can be reliably applied to HER2 prediction.

DSI integrates DTI, DKI, MAP, and NODDI to comprehensively characterize tissue microstructure. Mao et al. (14) applied multiparametric DSI in 170 breast cancers and identified NODDI-derived ICVF as an independent HER2 predictor, achieving AUCs of 0.700 and 0.721 in training and validation cohorts, respectively. Nevertheless, DSI requires specialized hardware and extensive post-processing, and the reproducibility of multiparametric fitting remains to be validated, limiting its current use to research settings rather than routine clinical practice.

Wang et al. (15) demonstrated that time-dependent diffusion histogram features achieved an AUC of 0.87 for distinguishing HER2-positive from HER2-negative disease, illustrating the complementary value of integrating multiparametric diffusion beyond single-parameter approaches. However, such integrated pipelines demand longer acquisitions, more complex processing, and greater reliance on harmonized protocols and rigorous validation, issues further discussed in the challenges section.

## 6 Dynamic contrast-enhanced MRI in HER2 prediction

DCE-MRI acquires serial images following gadolinium injection, enabling quantitative assessment of tissue perfusion, vascular permeability, and extracellular volume (24). Its high sensitivity for depicting tumor angiogenesis has established it as a key tool for molecular subtype assessment, including HER2-related phenotypes, and for monitoring response to neoadjuvant therapy.

### 6.1 Quantitative pharmacokinetic analysis

Pharmacokinetic modeling of DCE-MRI provides quantitative perfusion parameters: the volume transfer constant (Ktrans, reflecting vascular permeability and blood flow), the rate constant (Kep, describing contrast reflux from the extravascular extracellular space), and the extracellular volume fraction (Ve) (25). The Extended Tofts model is most widely used in HER2-related breast cancer studies, and parameters such as Vp (plasma volume fraction) may hold additional biological relevance in HER2-positive tumors due to their increased vascularity.

HER2 overexpression upregulates VEGF, promoting tumor angiogenesis (26); however, the resulting immature neovasculature elevates vascular permeability and interstitial fluid pressure (IFP) (27), which may impair the functional quality of tumor perfusion. Xie et al. (28) applied high-temporal-resolution DCE-MRI to breast cancer subtypes and found that minimum Kep was significantly lower in HER2-positive than HER2-negative tumors, suggesting impaired contrast reflux consistent with elevated IFP. These findings support the use of DCE-MRI pharmacokinetic parameters as non-invasive markers for HER2 assessment.

In radiomics studies, Li et al. (17) in 252 patients reported DCE radiomics training/testing AUCs of 0.742/0.776 for HER2-positive versus negative disease; a combined clinical–radiomics model reached testing AUC 0.915, supporting value beyond intratumoral features alone when integrated with clinical imaging. Liu et al. (19) assessed three-tier HER2 classification (zero/low/positive) in 400 patients, with the early post-contrast sequence (DCE2) contributing most to a combined AUC of 0.91. Yin et al. (29) further demonstrated that DCE-MRI radiomics could distinguish HER2-low from HER2-zero tumors with an AUC up to 0.892 (in the nomogram training cohort), supporting its potential to identify candidates for targeted therapy.

### 6.2 Semi-quantitative time-intensity curve (TIC) analysis

The time–intensity curve (TIC) classifies DCE-MRI contrast enhancement patterns into three types: Type I (persistent enhancement, typically benign), Type II (plateau), and Type III (washout, generally suggestive of malignancy). HER2-positive breast cancers more frequently exhibit a Type III curve (30), reflecting faster contrast washout driven by elevated vascular permeability and VEGF-mediated angiogenesis. HER2-overexpressing tumors more frequently show washout-type enhancement patterns (12).

Cheng et al. (30) demonstrated that radiomic features extracted from washout regions carry meaningful value for HER2 classification, consistent with the rapid early enhancement, steeper maximum slope, and shorter time-to-peak commonly observed in HER2-positive tumors. Liu et al. (19) further confirmed that radiomics models based on early-phase enhancement (DCE2) outperform those derived from delayed-phase images, highlighting the particular importance of early kinetic information.

Deep learning reduces subjectivity in TIC interpretation by directly learning dynamic contrast-enhancement features from imaging data, bypassing reliance on predefined patterns. In a multicenter study, Dai et al. (31) developed a DCE-MRI deep learning system achieving three-tier HER2 classification (zero, low, and positive) with internal testing AUCs of 0.776, 0.813, and 0.745, demonstrating that AI-driven analysis provides a scalable and objective approach to HER2 stratification.

Nonetheless, DCE-MRI has limitations for HER2 prediction. Quantitative parameters, including Ktrans and Ve, are sensitive to scanner variability, acquisition settings, and post-processing methods, limiting reproducibility across institutions (32), and they lack specificity for HER2 biology, as they may be elevated in tumors driven by other pro-angiogenic pathways. Semiquantitative TIC analysis is further constrained by the subjective distinction between Type II and Type III curves, introducing inter-reader variability. Moreover, most reported associations reflect correlation rather than direct mechanistic links between DCE-MRI parameters and HER2 expression.

## 7 Applications of metabolic and molecular MRI techniques in HER2 prediction

Metabolic and molecular MRI techniques, including MRS, CEST, and hyperpolarized ^13^C-MRI, have been investigated for breast cancer characterization. However, evidence for HER2-specific prediction remains limited and inconsistent.

### 7.1 Magnetic resonance spectroscopy (MRS)

Magnetic resonance spectroscopy (MRS) provides a non-invasive way to detect total choline (tCho), a marker closely related to membrane phospholipid metabolism. From a biologic standpoint, this is relevant because HER2 overexpression can activate downstream signaling pathways that enhance phospholipid turnover, potentially leading to higher tCho levels. In a 3 T study, Galati et al. (33) found that the presence of a tCho peak was strongly associated with established markers of tumor aggressiveness, including histologic grade 3 and high Ki-67 expression (*p* < 0.0001). Although the number of HER2-positive tumors in that cohort was relatively small, the findings suggest that tCho may have potential as a metabolic imaging marker for highly proliferative HER2-positive tumors.

A major limitation of tCho in clinical practice is its lack of specificity for HER2. In a study of 261 patients with early-stage, hormone receptor–positive, HER2-negative breast cancer, Kim et al. (34) found that higher tCho levels were significantly associated with 10-year disease-free survival and with late recurrence occurring 5–10 years after treatment. This suggests that elevated tCho is more likely to reflect overall tumor aggressiveness—such as increased membrane turnover and rapid cell proliferation—rather than metabolic changes unique to HER2 overexpression. As a result, when MRS is used to explore HER2 status non-invasively, the potential confounding effect of other highly proliferative subtypes, including HR-positive tumors, needs to be taken into account. Future work will likely need to move beyond tCho alone, either by combining it with other metabolic markers, such as phosphoethanolamine, or by incorporating it into multiparametric MRI models to improve the specificity of HER2 assessment.

### 7.2 Chemical exchange saturation transfer (CEST)

Amide proton transfer-weighted imaging (APTw), a form of chemical exchange saturation transfer imaging, reflects tissue protein content by measuring magnetization transfer from amide protons in mobile proteins and peptides. Its relationship with HER2 status, however, remains unclear. Xu et al. (35) reported that APTw-derived parameters could distinguish HER2-positive tumors from other molecular subtypes, suggesting potential value for subtype assessment. In contrast, Liu et al. (36) found that although APT imaging was associated with tumor grade, T stage, and proliferative activity, it did not show significant differences across HER2 groups. Taken together, these findings suggest that the association between APTw and HER2 is likely influenced by multiple biologic and technical factors, and its specificity for HER2 prediction remains to be established.

At present, the broader clinical use of CEST remains limited by unresolved issues in quantification and standardization. APT signals are highly sensitive to B0 and B1 field inhomogeneity, which can substantially affect measurement stability. In addition, conventional magnetization transfer ratio asymmetry analysis is vulnerable to interference from other effects, including the nuclear Overhauser effect, making interpretation less straightforward than it may appear. Another practical barrier is that post-processing tools on most commercial platforms are still not sufficiently mature for routine use. To move this technique closer to reliable HER2 assessment, future studies will need to standardize B0/B1 correction strategies and more clearly define whether APT provides subtype-specific information rather than reflecting broader tumor biology.

### 7.3 Hyperpolarized 13^C^-MRI and deuterium metabolic imaging (DMI)

Hyperpolarized 13^C^-MRI uses dynamic nuclear polarization to amplify the 13C signal by several orders of magnitude, making it possible to visualize tumor metabolism in real time. In breast cancer, Woitek et al. (37) showed that this technique could predict pathological complete response early during neoadjuvant chemotherapy by tracking pyruvate-to-lactate conversion, underscoring its potential for early treatment monitoring. Gallagher et al. (38) further demonstrated the feasibility of hyperpolarized ^13^C-MRI across different molecular breast cancer subtypes and, for the first time, revealed substantial intertumoral and intratumoral metabolic heterogeneity. Specifically, the lactate-to-pyruvate (LAC/PYR) signal ratio varied significantly among subtypes (0.021–0.473), with higher metabolic flux observed in aggressive phenotypes, including triple-negative and high-grade HER2-positive tumors.

Although metabolic imaging can capture important aspects of tumor aggressiveness, its role in more refined stratification—such as differentiating HER2-low from HER2-zero disease—remains largely exploratory. More prospective studies are still needed to determine how closely these metabolic imaging features correspond to HER2-related molecular pathways. By comparison, deuterium metabolic imaging (DMI) has the practical advantage of not requiring costly hyperpolarization hardware. However, its clinical application is currently limited by the inherently low gyromagnetic ratio of deuterium, which leads to a lower signal-to-noise ratio, as well as by insufficient spatial resolution for the assessment of small breast lesions. As a result, studies of DMI in breast cancer remain very limited at present.

In summary, the clinical value of metabolic and molecular MRI for HER2 stratification is not yet fully established. Even so, these techniques offer several complementary ways to assess tumor biology non-invasively. To move them closer to clinical use, greater technical standardization and clearer validation of their physiologic specificity will be essential. At present, they are better viewed as supportive tools that can provide whole-tumor metabolic information and help guide biopsy targeting, rather than as replacements for pathologic diagnosis.

## 8 Radiomics and artificial intelligence approaches

### 8.1 Evolution from conventional radiomics to multiparametric integration

Radiomics enables extraction of quantitative imaging features—including texture, shape, and signal intensity (39)—that capture tumor heterogeneity beyond visual assessment. An early systematic review by Davey et al. (40) confirmed that radiomics holds substantial potential for predicting breast cancer molecular subtypes, laying the groundwork for subsequent HER2-focused studies.

Integration of multiparametric MRI has driven recent advances. Liu et al. (19) combined DCE-MRI, DWI, and T2WI for three-tier HER2 classification (zero/low/positive), achieving AUCs of 0.91 and 0.88 in training and test sets, with the early post-contrast DCE2 phase contributing most. Li et al. (17) showed that adding 3-mm peritumoral features to intratumoral features improved performance, reaching AUCs of 0.923, 0.915, and 0.837 across three datasets. Liu et al. (41) further demonstrated that combining radiomics with conventional MRI features and clinical variables outperformed radiomics alone, supporting a multimodal approach.

Recent efforts have moved beyond binary HER2 classification to identify HER2-low disease. Yin et al. (29) achieved AUC up to 0.892 (nomogram training) for distinguishing HER2-low from HER2-zero tumors using DCE-MRI radiomics. Dai et al. (31) developed a multicenter deep learning model for three-category HER2 classification, with internal testing AUCs of 0.776, 0.813, and 0.745. Chen et al. (18) showed via SHAP analysis that early-phase DCE features contributed most to classification, with external validation AUCs of 0.757 (HER2-negative vs. positive) and 0.754 (HER2-zero vs. low). Moreover, Guo et al. (42) reported that imaging-predicted HER2 status correlated significantly with disease-free survival, suggesting that radiomics may capture clinically relevant tumor behavior beyond receptor expression. Despite encouraging AUC trends, the radiomics evidence should be interpreted with caution. Most studies were retrospective and conducted at a single center. PROBAST identified unclear participant selection in 24 of 34 studies and a high risk of bias in the analysis domain in 21 of 34 studies, while 10 of the 16 studies assessed with the RQS relied on internal validation only. Variability in segmentation and feature extraction pipelines further limits reproducibility, and the reported performance gains (17, 19) remain at the research stage rather than being ready for clinical application.

### 8.2 Optimization and performance enhancement of machine learning algorithms

Machine learning for HER2 assessment has evolved from binary to three-category stratification, aligning with newer therapies such as T-DXd. In a dual-center cohort of 1,015 patients, Zhao et al. (43) reported that support vector machines achieved the highest training AUC (0.86), whereas logistic regression generalized better with an external AUC of 0.66, highlighting algorithm-dependent cross-center performance. In a dual-center cohort of 737 patients, Chen et al. (18) applied a support vector machine for zero/low/positive classification, reporting external validation AUCs of 0.762 (HER2-negative versus HER2-positive) and 0.754 (HER2-zero versus HER2-low), with SHAP analysis indicating early-phase DCE features contributed most. These pipelines increasingly incorporate biologically meaningful imaging features, including high-temporal-resolution DCE kinetic parameters (28), rather than serving solely as statistical classifiers. Collectively, these findings support the use of multiparametric MRI–based machine learning for nuanced HER2 subclassification beyond binary prediction. Machine learning performance also depends on the study center and algorithm. For example, Zhao et al. (43) reported a training AUC of 0.86, which decreased to 0.66 in external validation. External validation was performed in only 9 of the 34 included studies; Chen et al. (18) reported external AUCs of 0.757 and 0.754. Retrospective study design, protocol heterogeneity, and model tuning on the same dataset increase the risk of overfitting. Therefore, external AUCs of approximately 0.66–0.84 provide a more realistic estimate of current generalizability than internal AUCs exceeding 0.90.

### 8.3 Deep learning: from CNNs to transformers

#### 8.3.1 Fundamental applications and architecture optimization of convolutional neural networks (CNNs)

Deep learning has advanced imaging-based HER2 prediction from reliance on hand-crafted features to end-to-end representation learning. Zhang et al. (44) were among the first to apply convolutional neural networks to DCE-MRI for molecular subtype prediction, using transfer learning to mitigate limited sample sizes and establishing an early foundation for subsequent breast MRI deep learning research.

Network architecture strongly influences model performance. Guo et al. (42) integrated conventional MRI radiomics with radiomic features derived from deep semantic segmentation in a dual-branch deep learning model. The model achieved validation AUCs of 0.763 for distinguishing HER2 overexpression from HER2-negative tumors and 0.750 for HER2-low versus HER2-zero classification, highlighting the advantage of multimodal feature fusion over single-source representations. Xie et al. (45) incorporated multiple DCE-MRI temporal phases for molecular subtype prediction, including HER2-enriched tumors, achieving external validation AUCs above 0.80 in two-center cohorts. Collectively, these studies suggest that deep learning models incorporating dynamic contrast-enhanced imaging features can support objective HER2 assessment. However, their cross-center generalizability remains limited by the predominance of internal validation and relatively small testing cohorts.

#### 8.3.2 Interpretability and multimodal fusion

Model interpretability remains a key barrier to clinical adoption of deep learning for HER2 assessment. In a dual-center cohort of 737 patients, Chen et al. (18) applied SHAP analysis to three-category HER2 MRI classification, quantifying individual feature contributions; early-phase dynamic contrast-enhanced variables exerted the greatest influence, with external validation AUCs of 0.762 and 0.754. Linking predictions to these inputs clarifies model behavior beyond black-box estimates and facilitates clinically relevant translation. Similar interpretability frameworks applied to large clinical cohorts have also identified ER and HER2 status as major predictors of response to neoadjuvant therapy (46). Although these models are not based on mpMRI, they complement rather than duplicate the MRI-focused evidence summarized above.

Among the included studies, integrating multiple MRI sequences with clinical variables within mpMRI frameworks consistently improved predictive accuracy and robustness, as demonstrated by Guo et al. (42) and Xie et al. (45). Likewise, Dai et al. (31) validated a multicenter deep learning framework based on DCE-MRI for three-tier HER2 classification, further highlighting the value of complementary imaging information in deep learning pipelines. Collectively, these findings support complementary multimodal designs for more robust and clinically interpretable HER2-related imaging models.

#### 8.3.3 Transformer architectures: technological frontier and future directions

Vision Transformers (ViTs) apply global self-attention to capture long-range spatial and temporal dependencies, in contrast to convolutional neural networks, which rely on local receptive fields. This holistic modeling approach may be well suited to multiparametric, multiphase MRI for HER2-related assessment, although dedicated validation in breast cancer remains limited.

#### 8.3.4 Summary of technological progress

Early single-parameter radiomics studies typically reported AUCs of approximately 0.71–0.78 (17). Integrating mpMRI improved performance to approximately 0.84–0.91 in internal or held-out testing (17) (19), whereas multicenter external validation more often yielded AUCs of 0.74–0.84 (18) and reached approximately 0.84 in the best-performing study (17). Deep learning and cross-modality fusion provide additional value (31), although AUCs above 0.90 have rarely been replicated in multicenter external validation. These improvements reflect the broader adoption of mpMRI, a shift from binary to clinically relevant three-tier HER2 classification, automated deep learning–based feature extraction replacing handcrafted features, attention mechanisms improving interpretability, and the emergence of Transformer architectures for time-resolved imaging. Because cohort composition, study design, validation strategy, sample size, MRI protocols, and reference standards differ across studies, the displayed AUC values are not directly comparable between studies and should not be interpreted as a performance ranking. (Figure 2).

## 9 Challenges and future perspectives

### 9.1 Methodological challenges and technical bottlenecks

Although current studies highlight the potential of mpMRI for HER2 assessment, several interrelated limitations constrain its clinical translation. Most evidence is retrospective, complicating control of selection bias and limiting confident extrapolation to prospective care. Imaging protocols, image quality, and post-processing vary across cohorts, obscuring expected routine performance, while many analyses rely on small samples, weakening statistical stability and cross-site generalizability. Inter-institutional heterogeneity remains substantial, as scanner hardware, acquisition settings, contrast administration, and analytic pipelines systematically shift radiomic and kinetic features, often leading to marked performance degradation when single-center models are externally tested. The Image Biomarker Standardisation Initiative (IBSI) framework (47) provides a foundation for standardizing radiomic feature calculation, yet achieving broader multicenter consistency remains challenging. Moreover, independent external validation is insufficient in many studies; internal validation alone can yield optimistic estimates, and clear roadmaps from model development to routine implementation are lacking. Such fragmentation complicates objective comparison of AUC values and undermines confidence in bedside deployment. Prospective, protocol-prespecified multicenter evaluation with transparent reporting therefore remains a priority, and harmonization, domain adaptation, or centralized quality control may mitigate site-specific shifts when rigorously validated.

Another vulnerability lies in the reference standard: immunohistochemical discrimination between HER2-low and HER2-zero exhibits substantial inter-observer variability (48), implying that models may learn inconsistently labeled targets, and reported accuracies require cautious interpretation. This instability is particularly relevant to HER2-low versus HER2-zero classification, where weak membrane staining (IHC 0 vs. 1+) is susceptible to inter-pathologist variability (48) and differences in antibody clones, detection systems, and laboratory protocols (4). Evolving ASCO/CAP criteria and intratumoral heterogeneity further compromise label consistency (3) (4). When IHC/ISH categories are used as the sole reference standard, radiomics and AI models may learn pathology-assigned labels rather than stable HER2 biology. Consequently, strong performance, particularly in distinguishing HER2-low from HER2-zero tumors, may partly reflect label noise rather than robust imaging biomarkers, and reported AUCs should therefore be interpreted with caution.

Finally, inconsistent adherence to TRIPOD recommendations (49) and established radiomics quality criteria further hampers reproducibility and impedes fair cross-study comparison.

### 9.2 Clinical positioning and future directions

Despite these limitations, mpMRI maintains a cautious yet meaningful role in HER2 assessment. Current evidence and practice support positioning mpMRI as a complement to, rather than a substitute for, biopsy-based pathology. Its principal strength lies in non-invasive, whole-tumor, repeatable imaging that mitigates single-site sampling bias while integrating morphologic, functional, and microenvironmental information, capturing heterogeneity that limited pathologic sampling may miss. mpMRI may inform pretreatment evaluation before neoadjuvant therapy and longitudinal monitoring during treatment. As HER2-low stratification refines targeted-therapy eligibility, mpMRI may help identify patients more likely to benefit and guide biopsy toward the most informative tumor regions, including when disease is multifocal. Along this trajectory, recent multicenter evidence suggests that mpMRI radiomics combined with deep learning can also support long-term survival prediction after neoadjuvant chemotherapy (50), indicating a broader shift from pretreatment HER2 stratification toward prognosis and treatment-outcome prediction. Although distinct from the focus of this review on non-invasive HER2 assessment, these findings highlight the expanding clinical applications of mpMRI-based AI. Figure 3 presents the assigned TRL levels based on the prespecified validation maturity criteria described in Section 2, emphasizing evidence maturity rather than ranking studies solely by AUC (Figure 3).

Future research should progress along five coordinated directions. First, larger prospective multicenter studies under routine care conditions are needed to validate performance and harmonize acquisition and analysis protocols. Second, greater rigor requires consistent IBSI-aligned radiomic feature extraction (47) and TRIPOD-conformant study reporting (49). Third, models should integrate imaging with pathology and genomic data within a multi-omics framework for HER2 evaluation. Fourth, federated learning and related strategies may ease data-sharing restrictions and enable scalable cross-center collaboration. Fifth, regulatory review and operational deployment standards must advance alongside technical innovation. Collectively, these efforts aim to reposition mpMRI from traditional diagnosis toward precision treatment decision support.

## 10 Conclusion

The integration of mpMRI with radiomics and artificial intelligence (AI) has markedly enhanced HER2 assessment in breast cancer. Key advances include the integration of peritumoral radiomics features (17), three-tier HER2 classification (19), and automated deep learning approaches based on DCE-MRI (31). Diagnostic performance has improved from approximately 0.71–0.78 in single-parameter studies to 0.84–0.91 with mpMRI integration in internal testing, although gains are more modest in multicenter external validation (17, 18).

Nevertheless, clinical translation is constrained by a more fundamental issue: the pathological reference standard itself is not fully stable. Substantial inter-observer variability in distinguishing HER2-low from HER2-zero (48) implies that imaging models may inadvertently predict diagnostic uncertainty rather than true biology. In this context, mpMRI should not be viewed as a replacement for pathology, but as a complementary tool providing a whole-tumor, dynamic, and multidimensional perspective. By capturing heterogeneity and microenvironmental features inaccessible to single-site biopsy, mpMRI can deliver clinically relevant information to guide more precise treatment decisions.

In the era of HER2-low–targeted therapy, mpMRI is shifting from a primarily diagnostic tool toward a role in treatment planning. Realizing this potential requires prospective multicenter validation, standardized workflows guided by frameworks such as IBSI and TRIPOD (47, 49), closer integration of imaging with pathology and genomics, and sustained cross-disciplinary collaboration.
