# Supplementary material for: Multiparametric MRI and artificial intelligence for non-invasive HER2 assessment in breast cancer: a comprehensive review
Source: Front Med (Lausanne). 2026 Aug 7;13:1893638. doi: 10.3389/fmed.2026.1893638 (PMC13493289; doi:10.3389/fmed.2026.1893638)
Supplement: Supplementary file 1 [file Table_1.pdf]

Supplementary Table S1. Complete database search strategies for PubMed, Web of Science, and Embase

Database	Date searched	Publication date limits	Interface / filters	Search strategy	Records (n)
PubMed	27 June 2026	1 January 2015 – 31 December 2025	PubMed (https://pubmed.ncbi.nlm.nih.gov/)	((breast cancer[MeSH Terms] OR breast neoplasms[MeSH Terms] OR breast carcinoma[Title/Abstract] OR breast tumor[Title/Abstract]) AND (HER2[Title/Abstract] OR ERBB2[Title/Abstract] OR HER-2[Title/Abstract] OR "human epidermal growth factor receptor 2"[Title/Abstract] OR HER2-low[Title/Abstract] OR HER2-positive[Title/Abstract] OR HER2-negative[Title/Abstract]) AND (magnetic resonance imaging[MeSH Terms] OR MRI[Title/Abstract] OR mpMRI[Title/Abstract] OR "multiparametric MRI"[Title/Abstract] OR "DCE-MRI"[Title/Abstract] OR "DWI"[Title/Abstract] OR "diffusion weighted imaging"[Title/Abstract] OR "dynamic contrast enhanced"[Title/Abstract] OR radiomics[Title/Abstract] OR "deep learning"[Title/Abstract] OR "machine learning"[Title/Abstract] OR "artificial intelligence"[Title/Abstract]) AND (predict*[Title/Abstract] OR classif*[Title/Abstract] OR discriminat*[Title/Abstract] OR assess*[Title/Abstract] OR noninvasive[Title/Abstract] OR "non-invasive"[Title/Abstract])) AND ("2015/01/01"[PDAT] : "2025/12/31"[PDAT])	870
Web of Science	27 June 2026	1 January 2015 – 31 December 2025	Web of Science Core Collection — Advanced Search	Web of Science Core Collection — Advanced Search (6 steps combined): Step 1: ((breast cancer OR breast neoplasm OR breast carcinoma OR breast tumor) Step 2: AND (HER2 OR ERBB2 OR HER-2 OR "human epidermal growth factor receptor 2" OR HER2-low OR HER2-positive OR HER2-negative) Step 3: AND (MRI OR "magnetic resonance imaging" OR mpMRI OR	207

				"multiparametric MRI" OR DCE-MRI OR DWI OR "diffusion weighted imaging" OR "dynamic contrast enhanced" OR radiomics OR "deep learning" OR "machine learning" OR "artificial intelligence")<br>Step 4: AND (predict* OR classif* OR discriminat* OR noninvasive OR "non-invasive")<br>Step 5: AND PY=(2015-2025)<br>Step 6: AND DT=(Article OR Early Access)	
Embase	27 June 2026	1 January 2015 – 31 December 2025	Embase — Advanced Search (Elsevier)	Embase — Advanced Search (5 query sets combined):<br>Set 1 (#1): 'breast cancer'/exp OR 'breast neoplasm'/exp OR 'breast neoplasms'/exp OR breast cancer:ab,ti OR breast carcinoma:ab,ti OR breast tumor:ab,ti<br>Set 2 (#2): 'HER2'/exp OR HER2:ab,ti OR ERBB2:ab,ti OR HER-2:ab,ti OR 'human epidermal growth factor receptor 2':ab,ti OR HER2-low:ab,ti OR HER2-positive:ab,ti OR HER2-negative:ab,ti<br>Set 3 (#3): 'magnetic resonance imaging'/exp OR MRI:ab,ti OR mpMRI:ab,ti OR 'multiparametric MRI':ab,ti OR 'DCE-MRI':ab,ti OR DWI:ab,ti OR 'diffusion weighted imaging':ab,ti OR 'dynamic contrast enhanced':ab,ti OR radiomics:ab,ti OR 'deep learning':ab,ti OR 'machine learning':ab,ti OR 'artificial intelligence':ab,ti<br>Set 4 (#4): predict*:ab,ti OR classif*:ab,ti OR discriminat*:ab,ti OR assess*:ab,ti OR noninvasive:ab,ti OR 'non-invasive':ab,ti<br>Combined: #1 AND #2 AND #3 AND #4 AND [2015-2025]/py<br>Post-search filter (result page sidebar): Publication type — Article; Article in press	691

Total records identified (before deduplication): 1,768 (870 + 207 + 691).

Notes: Duplicate records (n = 512) were removed in Zotero (identical DOI or identical title + first author + year), leaving 1,256 records for title/abstract screening. Source ledger: PRISMA_ledger.xlsx, sheet1.
