# Supplementary material for: Multiparametric MRI and artificial intelligence for non-invasive HER2 assessment in breast cancer: a comprehensive review
Source: Front Med (Lausanne). 2026 Aug 7;13:1893638. doi: 10.3389/fmed.2026.1893638 (PMC13493289; doi:10.3389/fmed.2026.1893638)
Supplement: Supplementary file 2 [file Table_3.pdf]

Supplementary Table 3. PROBAST traffic-light summary of included studies (n = 34)

Primary reviewer judgements. Green = low risk / low concern; Yellow = unclear; Red = high risk / high concern.

Study ID	First author, Year	Participants	Predictors	Outcome	Analysis	Overall applicability
1	Chen, 2024	Low	Low	Low	High	Low concern
2	Zhao, 2025	Unclear	Low	Low	High	Unclear
3	Wong, 2025	Unclear	Low	Low	Low	Low concern
4	Liu, 2025	Unclear	Low	Low	High	Unclear
5	Xu, 2022	Low	Low	Low	High	Low concern
6	Li, 2025	Low	Low	Low	Low	Low concern
7	Zhou, 2020	Unclear	Low	Low	Low	Low concern
8	Hu, 2025	Unclear	Low	Low	High	Unclear
9	Zhou, 2025	Unclear	High	Low	High	Unclear
10	Lu, 2020	Unclear	Low	Low	High	Unclear
11	Jiang, 2019	Low	High	Low	High	Low concern
12	Yang, 2025	Unclear	Low	Low	High	Unclear
13	Song, 2021	Low	High	Low	Low	Unclear
14	Qiu, 2025	Unclear	Low	Low	Low	Low concern
15	Li, 2025	Low	Low	Low	High	Low concern
16	Fang, 2022	Unclear	Low	Low	High	Unclear
17	Mao, 2022	Unclear	Low	Low	High	Unclear
18	Shen, 2025	Low	Low	Low	High	Low concern
19	Liu, 2025	Unclear	Low	Low	High	Unclear
20	Zhan, 2024	Unclear	Low	Low	High	Unclear
21	Lin, 2025	Unclear	Low	Low	High	Unclear
22	Chen, 2025	Low	Low	Low	High	Low concern
23	Huang, 2024	Unclear	Low	Low	High	Unclear
24	Zhao, 2024	Low	High	Low	High	Low concern
25	Kang, 2022	Low	High	Low	High	Low concern
26	Li, 2025	Unclear	Low	Low	Low	Low concern
27	Yin, 2024	Unclear	Low	Low	Low	Low concern
28	Cheng, 2025	Unclear	High	Low	Low	Unclear
29	Dai, 2025	Unclear	Low	Low	Low	Low concern
30	Liu, 2024	Unclear	Low	Low	Low	Low concern
31	Zhao, 2025	Unclear	Low	Low	Low	Low concern
32	Zhang, 2020	Unclear	Low	Low	Low	Low concern
33	Guo, 2023	Unclear	Low	Low	Low	Low concern
34	Xie, 2024	Unclear	Low	Low	High	Unclear

Summary counts

Domain / overall	Low / low concern	High / high concern	Unclear
Participants	10	0	24
Predictors	28	6	0
Outcome	34	0	0
Analysis	13	21	0
Overall applicability	19	0	15
