# Supplementary material for: Multiparametric MRI and artificial intelligence for non-invasive HER2 assessment in breast cancer: a comprehensive review
Source: Front Med (Lausanne). 2026 Aug 7;13:1893638. doi: 10.3389/fmed.2026.1893638 (PMC13493289; doi:10.3389/fmed.2026.1893638)
Supplement: Supplementary file 3 [file Table_4.pdf]

Supplementary Table S4. Cross-reference of included studies for Figures 2 and 3 (n = 34)

Study ID	First author, year	DOI	Modality / technique	Highest AUC	TRL	Fig. 3	Validation
1	Chen, 2024	10.1186/s13058-024-01921-7	Radiomics — Habitat-based MRI radiomics	0.940	7	Yes	Multi-center, 2C
2	Zhao, 2025	10.1186/s40644-025-00981-y	Radiomics — DCE + IVIM radiomics	0.903	5	Yes	Internal, 1C
3	Wong, 2025	10.1186/s13058-025-02118-2	Deep Learning — AI foundation model (DCE-MRI)	0.857	7	Yes	External, multiC
4	Liu, 2025	10.1038/s41598-025-31322-5	T2WI / Synthetic MRI — Synthetic MRI (T2 quant.)	0.876	7	Yes	Internal + External, 2C
5	Xu, 2022	10.1186/s12885-022-09967-6	Radiomics — Clinicoradiomic nomogram	0.945	5	Yes	Internal, 1C
6	Li, 2025	10.1186/s12885-025-14198-6	Machine Learning — AutoML ADC radiomics	0.820	5	Yes	Internal, 1C
7	Zhou, 2020	10.1016/j.acra.2020.05.040	Radiomics — mpMRI radiomics (T2 + DCE)	0.860	5	Yes	Internal, 1C
8	Hu, 2025	10.1186/s40001-024-02188-6	DCE-MRI — Quantitative DCE (perfusion)	0.850	5	Yes	Internal, 1C
9	Zhou, 2025	10.1002/jmri.29447	Machine Learning — BI-RADS features + ML	0.860	5	Yes	Internal, 1C
10	Lu, 2020	10.3389/fonc.2020.00543	Radiomics — DCE texture (subregions)	0.929	5	Yes	Internal, 1C
11	Jiang, 2019	10.3389/fonc.2019.00242	Radiomics — DCE texture analysis	0.865	5	Yes	Internal, 1C
12	Yang, 2025	10.3389/fonc.2025.1709170	T2WI / Synthetic MRI — SyMRI + MUSE-DWI	0.940	5	Yes	Internal, 1C
13	Song, 2021	10.3389/fonc.2021.675160	Radiomics — DCE kinetic texture maps	0.920	5	Yes	Internal, 1C
14	Qiu, 2025	10.3389/fonc.2025.1593033	Deep Learning — Multimodal MRI + DL nomogram	0.940	7	Yes	Multi-center
15	Li, 2025	10.3389/fonc.2025.1531553	Radiomics — mpMRI intra+peritumoral radiomics	0.923	7	Yes	Internal + External, 2C
16	Fang, 2022	10.3389/fonc.2022.922185	Radiomics — Clinical-radiomics nomogram	0.870	7	Yes	Multi-center, 2C
17	Mao, 2022	10.3389/fonc.2022.817070	Advanced DWI — DSI (NODDI-ICVF)	0.721	4	Yes	Split-sample, 1C
18	Shen, 2025	10.3390/tomography11030031	Conventional MRI — Qualitative + DKI histogram	0.903	5	Yes	Internal, 1C
19	Liu, 2025	10.1016/j.crad.2025.106955	Conventional MRI — Conventional MRI + clinical	0.874	5	Yes	Internal, 1C
20	Zhan, 2024	10.1016/j.ejrad.2024.111573	T2WI / Synthetic MRI — Synthetic MRI (PDe param.)	0.849	5	Yes	Internal, 1C
21	Lin, 2025	10.1002/jmri.70074	Advanced DWI — Time-	0.870	4	Yes	Prospective, 1C

			dependent DWI (t-dMRI)				
22	Chen, 2025	10.2214/ajr.24.31717	Radiomics — mpMRI radiomics + SVM (3-class)	0.757	7	Yes	External, 2C
23	Huang, 2024	10.1093/bjr/tqae124	Machine Learning — XGBoost + MRI features	0.787	5	Yes	Internal, 1C
24	Zhao, 2024	10.1016/j.acra.2024.11.011	Advanced DWI — IVIM + DCE combined (3-class)	0.860	5	Yes	Internal, 1C
25	Kang, 2022	10.1002/jmri.27999	Advanced DWI — DKI (mean kurtosis)	0.781	3	Yes	Internal, 1C
26	Li, 2025	10.1177/02841851251319110	Radiomics — mpMRI LASSO + LR (3-class)	0.910	5	Yes	Internal, 1C
27	Yin, 2024	10.3389/fonc.2024.1385352	DCE-MRI — DCE radiomics (low vs zero)	0.892	5	Yes	Internal, 1C
28	Cheng, 2025	10.3389/fmolb.2025.1635296	DCE-MRI — DCE washout radiomics	0.876	5	Yes	Internal, 1C
29	Dai, 2025	10.1002/jmri.29670	Deep Learning — ResNetGN auto-seg DCE (3-cls)	0.820	8	Yes	External, multiC
30	Liu, 2024	10.1016/j.acra.2024.07.048	Radiomics — cMRI + radiomics (3-class)	0.842	5	Yes	Internal, 1C
31	Zhao, 2025	10.3389/fcell.2025.1669651	Machine Learning — SVM / LR / ANN (multi-class)	0.870	8	Yes	External, multiC
32	Zhang, 2020	10.1007/s00330-020-07274-x	Deep Learning — CNN / CLSTM (DCE subtypes)	N/A (accuracy only; not in Figure 3 bubble plot)	6	No	Internal + transfer, 2C
33	Guo, 2023	10.1007/s00330-023-09990-6	Deep Learning — DL radiomics (DLR signature)	0.868	5	Yes	Internal, 1C
34	Xie, 2024	10.1016/j.acra.2024.03.002	Deep Learning — MBCNN multi-phase DCE	0.850	7	Yes	Multi-center

Notes: Study IDs match Sheet 6 and Figure 2/3 labels (not reference ordinals). ID 32 omitted from Figure 3 bubble plot (accuracy only). Source: fig2_curated_34.json, fig3_curated_34.json, PRISMA_ledger.xlsx Sheet 6.
